# Syndemic Risk Classes and Substance Use Problems among Adults in High-Risk Urban Areas: A Latent Class Analysis

**DOI:** 10.3389/fpubh.2017.00237

**Published:** 2017-09-07

**Authors:** Charles M. Cleland, Stephanie T. Lanza, Sara A. Vasilenko, Marya Gwadz

**Affiliations:** ^1^Center for Drug Use and HIV Research, New York University Meyers College of Nursing, New York, NY, United States; ^2^Department of Biobehavioral Health, The Pennsylvania State University, University Park, PA, United States

**Keywords:** syndemics, resilience, risk, substance use problem, latent class analysis

## Abstract

Substance use problems tend to co-occur with risk factors that are especially prevalent in urban communities with high rates of poverty. The present study draws on Syndemics Theory to understand profiles of risk and resilience and their associations with substance use problems in a population at risk for adverse outcomes. African-American/Black and Hispanic heterosexual adults (*N* = 2,853) were recruited by respondent-driven sampling from an urban area with elevated poverty rates, and completed a structured assessment battery covering sociodemographics, syndemic factors (that is, multiple, co-occurring risk factors), and substance use. More than one-third of participants (36%) met criteria for either an alcohol or a drug problem in the past year. Latent class analysis identified profiles of risk and resilience, separately for women and men, which were associated with the probability of a substance use problem. Almost a third of women (27%) and 38% of men had lower risk profiles—patterns of resilience not apparent in other types of analyses. Profiles with more risk and fewer resilience factors were associated with an increased probability of substance use problems, but profiles with fewer risk and more resilience factors had rates of substance use problems that were very similar to the general adult population. Relative to the lowest risk profile, profiles with the most risk and fewest resilience factors were associated with increased odds of a substance use problem for both women [adjusted odds ratio (aOR) = 8.50; 95% CI: 3.85–18.74] and men (aOR = 11.68; 95% CI: 6.91–19.74). Addressing syndemic factors in substance use treatment and prevention may yield improved outcomes.

## Introduction

Substance use problems tend to co-occur with other serious risk factors that are especially prevalent in communities with high rates of poverty, and where African-American/Black and Hispanic populations are over-represented in comparison to the general underlying population (1, 2). These risk factors may be related in complex ways, occurring together at the same time, in the same person, or in the same community. Methods are needed to yield new insights into these complex patterns of co-occurring risk factors, by identifying core patterns of vulnerabilities and assets. This study applies latent class analysis (LCA) to understand patterns of risk and resilience among adults in communities considered high-risk, focusing on heterosexuals, and then describes associations among latent classes and recent substance use problems.

Recently, we conducted a set of community-based studies focused on African-American/Black and Hispanic heterosexuals considered at high risk for HIV infection by virtue of their geographical and social connections to urban areas with elevated rates of both poverty and prevalent HIV, called “high-risk areas” (3–6). In addition to elevated risk for HIV infection, this population evidences a high prevalence of numerous, diverse contextual and psychosocial risk factors for other adverse health and behavioral outcomes in comparison to the general population. In particular, modest levels of educational achievement and health literacy, and high rates of unemployment, homelessness, incarceration, episodes of extreme poverty, and clinically significant depressive symptoms are common among high-risk heterosexuals, along with only moderate levels of emotional and instrumental support (3–5, 7, 8). Moreover, the population experiences heightened rates of problematic alcohol and/or drug use (3–5, 7). Of concern, substance use problems have grave negative consequences for physical and mental health and well-being (9), and are highly stigmatizing, thus interfering with important social relationships, employment, and involvement in health-care settings (10, 11). Further, poverty often increases the harms associated with substance use (11, 12). While it is well known that risk factors for adverse health outcomes tend to co-occur (2, 13), little is known about specific patterns of risk and resilience in this large population at high risk for poor outcomes, including regarding the potential associations of risk and protective factors with substance use problems. Grounded in this literature, we selected the following potential risk and potentially protective background and psychosocial factors for study: education level, experiences of extreme poverty, employment status, history of homelessness, history of incarceration, health literacy, depression, emotional support, and instrumental support (3–5).

Despite the overall pattern of risk and vulnerability among heterosexuals in high-risk areas (referred to as “H-HRA”) described above, there is growing awareness that treating risk factors as separate indices creates an incomplete description of the population, and further, may obscure indications of resilience. Indeed, substantial variability is evident in rates of the types of risk factors described above; for example, most are unemployed, a risk factor, but a substantial proportion have achieved at least a high school (HS) diploma, an indication of resilience (3–5, 7). Syndemics Theory describes how two or more conditions cluster together by person, place, or time interacts synergistically, thereby collectively increasing disease burden (14, 15). Indeed, Syndemics Theory posits that these types of problems and risk factors are, in fact, best both understood and addressed collectively (16, 17). Syndemics Theory was first used to describe the confluence of substance abuse, violence, and AIDS (18), and more recently, the theory has been applied to understand risk among populations such as men who have sex with men (19, 20), and persons living with HIV (14, 21). However, less research has applied Syndemics Theory among high-risk heterosexuals (15, 22). Yet a better understanding of clusters or patterns of risk and resilience in this population of H-HRA has potential to the needs of this population, thereby improving the efficiency and efficacy of screening, outreach, and treatment programs and initiatives. This emphasis on substance use screening, outreach, and treatment is consistent with the US Surgeon General’s call for an expanded public health approach to substance use problems in the US (23).

Latent class analysis (24) is a statistical method that uncovers latent subgroups, or “classes,” defined by distinct response patterns on multiple relevant risk factors. LCA is well suited to examining the multidimensional, synergistic nature of syndemics because it can assess specific combinations of multiple risk factors simultaneously. This approach has been applied to study syndemics among low-income women (25), people living with HIV and a history of injection drug use (26), exotic dancers (27), and in a national study on alcohol and related conditions (28). Robinson and colleagues used LCA to identify patterns of substance use, mental illness, and familial conflict, which were related to viral suppression and acute care utilization (26). These applications of LCA show its flexibility and utility in identifying patterns of vulnerability and resilience and relating those patterns to important health outcomes.

In the present study, we employ LCA to examine latent classes of risk factors among H-HRA, and explore potential associations between these latent classes and substance use problems. Age was included as a covariate in LCA since risk factors and the probability of a substance use problem are likely to increase with age. In addition, we examine profiles of risk separately for men and women, because men and women interact with dissimilar health-care systems and institutions (e.g., women receive prenatal services and men are more likely to be incarcerated) (3, 29). Furthermore, as Ostrach and Singer have posited (30), women may be particularly vulnerable to health threats compared to men, due to sociopolitical environmental factors and multiple pathways of risk. While men evidence higher rates of substance use problems than women, women may face more serious consequences related to use, such as sexual assault or reproductive problems (31, 32). Considering the dearth of studies on H-HRA, we do not present formal hypotheses in the present paper, but instead use LCA to explore classes of risk, and their associations with substance use problems, separately for men and women. Study findings will be of interest to program planners, policy makers, interventionists, and other stakeholders working toward the prevention and treatment of substance use problems among H-HRA, and those invested in urban health generally.

## Materials and Methods

### Participants and Procedures

Data for the present study were drawn from a baseline assessment conducted as part of a larger study testing approaches for identifying undiagnosed HIV infection among African-American/Black and Hispanic H-HRA (5). Participants were recruited in 2012–2015 using respondent-driven sampling (RDS) (33, 34). RDS is a peer-to-peer recruitment method designed to reach deep into hidden or at-risk populations, by engaging more isolated or vulnerable network members who may not be present in traditional institutional or social venues and/or not typically willing to engage in research (35). In practice, RDS begins with direct recruitment by staff of “initial seeds” in public venues, who then recruit their peers until sample size goals are met (RDS procedures are described in more detail below). The study was approved by the Institutional Review Board of the New York University School of Medicine. Participants gave signed informed consent for study activities.

#### Study Setting

The study was conducted in a well-defined, high-risk area in central Brooklyn in New York City. To create the boundaries of the high-risk area, an index was calculated for each zip code in Brooklyn by standardizing census-based poverty levels and case surveillance-based heterosexual HIV prevalence to overall levels in Brooklyn, and then ranked. Next, a core high-risk area was identified using the local indicators of spatial association procedure (comprised seven zip codes). To reduce artificial restrictions on RDS recruitment chains, a larger high-risk area was then demarcated, adding remaining zip codes in the top 50% of the empirical distribution of the index (12 additional zip codes). A study field site was located in the core high-risk area, as was recruitment of the initial seeds who started the RDS recruitment chains. However, recruitment of peers during RDS could extend to the larger high-risk area. Procedures used to create the high-risk area are described in more detail elsewhere (5).

#### Eligibility Criteria

The main study eligibility criteria were: age 18–60 years, sexually active with ≥1 opposite sex partner in the past year, resides in the high-risk area, African-American/Black or Hispanic racial/ethnic backgrounds (because the larger study focused on the populations at highest risk for HIV), comprehends English or Spanish, and not actively psychotic based on a standard screening instrument. Additional inclusion criteria for initial seeds for RDS included negative/unknown HIV status and residence in the core high-risk area (not the larger high-risk area). RDS peers could have a previous HIV diagnosis, in keeping with the goals of the larger study, but these HIV-infected participants are not included in the present study, because their risk factors, service use patterns, substance use patterns, and health outcomes vary substantially from their HIV-negative peers (36–38).

#### Recruitment

For the present study, a total of 107 initial seeds, selected to vary in age, gender, and race/ethnicity, were directly recruited by study staff in 2012–2014 from public and street venues within the core high-risk area, and then enrolled into the study. Each initial seed could start a recruitment chain by recruiting three to five peers, and these peers then enrolled into the study and recruited their own peers until sample size goals were met. The average number of “waves” on these recruitment chains was 7 (range 1–26 waves/chain), leading to a sample size of 3,002. Overall, 66% of participants recruited at least one peer (of these, 47% of initial seeds recruited ≥1 peer). Both seeds and peers are included as participants in the present study, but some participants were excluded if they were missing data on age (*n* = 3), race/ethnicity (*n* = 1), or one or more of the nine latent class indicator variables (*n* = 145). Thus, the sample size for the present study was 2,853 participants.

#### Enrollment and Assessment

Apart from initial seeds, participants were recruited by peers and presented to the study field site with a coded recruitment coupon linking them back to the recruiter. Potential participants, both initial seeds and peers, provided verbal informed consent and were screened for eligibility with a brief structured assessment (10 min). Those found eligible provided signed informed consent for remaining study activities and then completed a structured baseline interview. The interview lasted 60–90 min and was administered by trained staff on computers using an audio, computer-assisted self-interviewing program (39). Participants received compensation of $15 for the screening and $30 for the baseline interview, as well as funds for two-way local public transportation. The recruiter received $15 for each peer referred and found eligible.

### Measures

The measures used in the present study were drawn from the harmonized instruments used for the set of “Seek, Test, Treat, and Retain” studies sponsored by the National Institute on Drug Abuse (NIDA) at the National Institutes of Health (40). These measures have been used in past studies with H-HRA and similar vulnerable populations, and are described below. Cronbach’s alpha is provided for scales where appropriate.

#### Sociodemographic and Background Factors

Using a structured NIDA-harmonized instrument (41), we assessed age (in years), race/ethnicity (African-American/Black, Latino/Hispanic), and sex (male/female). The remaining sociodemographic indices were coded to reflect the predominant categories and were coded as yes/no, with the “yes” value presented in Table 1 for parsimony. These included marital status (married or living as married or in a long-term relationship), income from government benefits, and whether had any health insurance.

**Table 1:** Sociodemographic characteristics and syndemic factors investigated as risk factors for substance use problems.

	Female (*n* = 1,203)	Male (*n* = 1,650)	Total (*n* = 2,853)	*p*-Value^a^

	Mean (SD)/%	Mean (SD)/%	Mean (SD)/%	
**Background characteristics**				
Age in years	37.2 (12.0)	39.0 (12.2)	38.2 (12.2)	<0.001
African-American, not Hispanic	77.6	72.5	74.7	0.002
Latino/Hispanic	22.3	27.4	25.2	0.003
Married or in long-term relationship	41.3	29.5	34.5	<0.001
Portion of income includes gov’t benefits	34.1	28.2	30.7	0.001
Currently has health insurance	90.5	81.1	85.1	<0.001
**Syndemic factors**				
High school diploma or equivalent or higher	60.3	63.4	62.1	0.101
Not engaged in full-time or part-time work currently	62.3	72.8	68.4	<0.001
Ever homeless	55.8	58.2	57.2	0.251
Currently homeless	17.9	23.3	21.0	<0.001
Ever been incarcerated for >24 h	41.4	74.4	60.5	<0.001
Incarcerated in the past year for >24 h	11.4	30.3	22.3	<0.001
Unable to pay for necessities in past year	40.6	46.8	44.2	0.001
Low health literacy	14.6	16.7	15.8	0.133
Clinically significant symptoms of depression (CESD)	31.2	28.7	29.8	0.159
Emotional support	70.7	62.2	65.8	<0.001
Instrumental support	59.9	53.3	56.1	<0.001
**Substance use**				
Any drug use in the past month	26.6	36.4	32.3	<0.001
Drug use frequency past month (0–8)	1.0 (2.1)	1.5 (2.5)	1.3 (2.4)	<0.001
Ever injected drugs not for a medical reason	5.7	10.8	8.7	<0.001
Injected drugs in the past month	0.5	1.8	1.3	0.002
Meets AUDIT criterion for alcohol problem—past year	24.9	25.3	25.1	0.827
Meets TCU criterion for drug problem—past year	14.0	25.1	20.4	<0.001
**Primary outcome**				
Meets criteria for substance use problem—past year	32.1	38.4	35.7	<0.001

*^a^Females and males were compared by independent samples t-tests or Fisher’s exact test*.

#### Syndemic Risk Factors

The nine syndemic factors, described next, included education level, inability to pay for basic necessities, work status, history of homelessness, history of incarceration, health literacy, depression, emotional support, and instrumental support. Items were coded such that higher values indicate greater risk. Using a structured NIDA-harmonized instrument (42), we assessed *education level*, namely whether the participant has achieved at least a HS diploma or passed a *General Educational Development* (*GED*) test (or more; coded 1) or has no HS diploma and has not completed a GED test (coded 2). Participants indicated whether they were unable to pay for *necessities* monthly or more often (coded 1 for no, 2 for yes); this serves as an indicator of extreme poverty. Current *work status* was reported, indicating whether the participant is currently employed part- or full-time, retired, a homemaker, or a student (coded 1) or unemployed, disabled, laid off, or incarcerated (coded 2). Individuals reported whether they ever had been *homeless* in their lifetime (coded 1 for no, 2 for yes). They also reported whether they ever had been *incarcerated* in prison or jail in their lifetime (coded 1 for no, 2 for yes). *Health literacy* was assessed by a scale comprising three items coded on five-point scales (e.g., “feel confident filling out medical forms by yourself”; α = 0.55); scale scores greater than 3 indicated higher health literacy (coded 1) and scores of 3 or lower indicated low health literacy (coded 2). An item also was included that indicated whether the participant’s *depressive symptoms* were at a clinically significant level or severe level over the past week [20-item CES-D; α = 0.80; (43)]. The composite depression score was calculated and values below 16 were coded as no depression (coded 1); values from 16 to 21 were coded as clinically depressed but not severe (coded 2); values of 22 or greater indicated severe depression (coded 3). *Emotional support* was assessed with a single 5-category item (“Someone to love and make you feel wanted”); the indicator for LCA was coded 1 for most or all of the time (higher support) and 2 for some, a little, or none of the time (low support). *Instrumental support* was assessed as a scale score based on four 5-category items (e.g., “Someone to help with daily chores if you were sick”; α = 0.88). Mean scores of 3 or greater indicated higher (coded 1) and mean scores less than 3 indicated low instrumental support (coded 2).

#### Substance Use Problems

Using recognized screening measures and established cutoff values for problematic levels of use, we assessed drug use and alcohol problems. The presence of drug use problems in the past year was based on 9 items from the TCU Drug Screen II [Cronbach’s alpha (α) = 0.91] (44), which has high sensitivity and specificity in the detection of drug abuse or dependence disorder (45). One point was assigned for an affirmative response to each of the nine items, yielding a total score ranging from 0 to 9. Participants with scores less than 3 were coded 1 for no drug use problem, and those with scores of 3 or higher were coded 2 for yes (46). The presence of alcohol problems in the past year was assessed based on 10 items from the AUDIT (α = 0.89) (47), which has high sensitivity and specificity in the detection of hazardous or harmful alcohol consumption (47, 48). Individual items were scored from 0 to 4 and summed, yielding a total score ranging from 0 to 40. Women with total scores less than 5 and men with scores less than 8 were coded 1 for no alcohol problems. Women with total scores of 5 or above and men with scores of 8 or above were coded 2 for yes for alcohol problems (49). A single indicator of substance use problem was then created (coded 1 for neither problem, 2 for one or both problems).

### Statistical Analysis

Analyses were conducted separately for men and women to allow sex to fully moderate the syndemic class structure. Separate analyses were conducted for each gender because both syndemic indicators and substance use problems have important gender differences (50–53). The large sample size was an opportunity to examine syndemics and their relations with substance use problems separately for women and men. In general, conducting LCA separately by gender allows this variable to fully moderate the complex model. That is, the number of classes, class sizes, measurement parameters, and associations of classes with covariates all can vary across gender.

For each gender, models with one through seven classes were estimated and compared and the optimal model selected based on adjusted likelihood ratio tests (54, 55). Information criteria (e.g., Akaike Information Criterion and Bayesian Information Criterion), which compare relative fit of competing models with penalties for complexity, entropy (degree to which only one latent class is highly probable for each individual), and chi-squared tests were reported for additional detail on model fit. Age was then included as a covariate to determine, for men and women separately, whether it was associated with latent class membership. Finally, latent class membership was used to predict the binary outcome reflecting any alcohol and/or drug use problem. Associations between age and latent class membership were estimated using multinomial logistic regression. Associations between latent class membership and a substance use problem were estimated using binary logistic regression. An inclusive classify-analyze approach was used, in which the optimal LCA models and assignment of individuals to classes were carried out with covariates included in the models (56). Multiple (50) pseudo-class draws were used to assign individuals to latent classes using posterior probabilities from the inclusive LCA. Latent classes with the lowest probability of endorsing risks were coded as reference categories in the outcome analyses. Estimates of prevalence for a single variable from an RDS are often weighted by network size and patterns of recruitment. However, it is common not to use RDS weights in multivariate analysis, and all reported analyses were unweighted. All latent class models were fit using Mplus (57, 58); other analyses were conducted using the R statistical computing environment (59).

## Results

The prevalence of each demographic and study variable is presented for men and women, along with corresponding tests for gender differences, in Table 1. Approximately 42% of participants were women. Excluding seed participants, 66.0% of male participants were recruited by a male recruiter, and 54.5% of female participants were recruited by a female recruiter. Thus, a female recruiter recruited 34% of male participants and a male recruiter recruited 45.5% of female participants.

Overall (i.e., across both women and men), 1,019 out of 2,853 (35.7%) were identified as having an alcohol and/or drug use problem; the rates for men (38.4%) and women (32.1%) were significantly different (OR = 1.32). On average, men were about 2 years older than women, were more likely to be Hispanic, and were less likely to be in a long-term relationship. Compared with women, men also were less likely to have health insurance, government benefits, full-time or part-time work, emotional support, and instrumental support. Men were more likely than women to have current homelessness, a history of incarceration, an inability to pay for necessities, drug use in the past month, a history of injection drug use, and injection drug use in the past month.

### Syndemic Class Membership

Model selection was conducted separately for women and men. For each sex, models with from one to seven classes were compared (see Table 2). The AIC always favored more complex models for both women and men, and so was not used in model selection. The three-class model was selected for women; parameter estimates are presented in Table 3. The three-class model for women was identified as best by the Vuong–Lo–Mendell–Rubin likelihood ratio tests of nested models. BIC was lowest for the two-class model among women, but the three-class solution was preferred as it differentiated women with only historical risks from women with lower probabilities of both current and historical syndemic risk factors. In the three-class solution, Class 1 was labeled *Assets and Few Historical Risks* (27.1% of women) and includes women with low probabilities of endorsing most risks. Risks were not completely absent in this low risk class, as women in this class had modest probabilities of endorsing basic needs not met (0.268), low education (0.315), and current unemployment (0.418). On the other hand, women in the *Assets and Few Historical Risks* class were likely to have assets such a higher education level (0.685), employment (0.582), and social support (0.911), and an absence of historical risk factors such as homelessness (0.814 never homeless). Class 2 was labeled *Historical Risk/Assets* (34.4%); these women were likely to have histories of homelessness (0.700) and incarceration (0.571), and were likely to be unemployed (0.635), but were otherwise similar to women in the *Assets and Few Historical Risks* class. Class 3 was labeled *Low Resources and Support* (38.4%); these women were more likely than women in other classes to have unmet basic needs (0.623), clinically significant (0.278) or severe (0.381) depression, low education (0.546), low emotional support (0.654), low health literacy (0.255), low instrumental support (0.756), and current unemployment (0.756).

**Table 2:** Model selection information.

Gender	Number of classes	*G*^2^	Degrees of freedom	Parameters	AIC	BIC	Entropy	VLMR^b^ *p*
Females (*N* = 1,203)	1	2,135.44	1,713	12	15,656.02	15,717.13	–	–
	2	1,410.70	1,702	25	14,938.61	15,065.92	0.69	<0.0001
	3^a^	1,328.25	1,689	38	14,882.16	15,075.68	0.62	0.0399
	4	1,249.03	1,676	51	14,828.94	15,088.66	0.64	0.9997
	5	1,195.25	1,663	64	14,801.16	15,127.08	0.67	0.0524
	6	1,165.16	1,650	77	14,797.07	15,189.20	0.73	0.3580
	7	1,118.58	1,635	90	14,795.58	15,253.91	0.73	1.0000

Males (*N* = 1,650)	1	2,542.72	1,715	12	22,058.11	22,123.01	–	–
	2	1,745.66	1,702	25	21,287.05	21,422.26	0.61	<0.0001
	3^a^	1,568.76	1,689	38	21,136.15	21,341.68	0.71	0.0174
	4	1,445.77	1,676	51	21,039.15	21,314.99	0.63	0.2576
	5	1,350.71	1,663	64	20,970.10	21,316.24	0.64	0.1671
	6	1,289.11	1,650	77	20,934.49	21,350.95	0.65	0.9157
	7	1,257.38	1,637	90	20,928.77	21,415.54	0.68	0.8643

*^a^Selected model*.

*^b^Vuong–Lo–Mendell–Rubin likelihood ratio test comparing each model to the model with one fewer classes (54, 55)*.

**Table 3:** Three syndemic classes among high-risk heterosexuals: class prevalences and item-response probabilities.

	Female			Males		
Latent class indicator	Class 1 (*n* = 326; 27.1%)	Class 2 (*n* = 414; 34.4%)	Class 3 (*n* = 462; 38.4%)	Class 1 (*n* = 627; 38.0%)	Class 2 (*n* = 500; 30.3%)	Class 3 (*n* = 523; 31.7%)
		
Label	Assets and few historical risks	Historical risk/assets	Low resources and support	Personal assets	Low resources/social assets	Low resources and support
Basic needs met	**0.732**	**0.726**	0.377	**0.758**	0.383	0.402
Basic needs not met	0.268	0.274	**0.623**	0.242	**0.617**	**0.598**
Education low	0.315	0.293	**0.546**	0.256	0.447	0.421
Education not low	**0.685**	**0.707**	0.454	**0.744**	**0.553**	**0.579**
Health literacy low	0.098	0.064	0.255	0.070	0.233	0.220
Health literacy not low	**0.902**	**0.936**	**0.745**	**0.930**	**0.767**	**0.780**
Work employed	**0.582**	0.365	0.244	0.428	0.138	0.211
Work unemployed	0.418	**0.635**	**0.756**	**0.572**	**0.862**	**0.789**
Instrumental support low	0.150	0.202	**0.756**	0.231	0.373	**0.839**
Instrumental support not low	**0.850**	**0.798**	0.244	**0.769**	**0.627**	0.161
Emotional support low	0.089	0.050	**0.654**	0.159	0.000	**1.000**
Emotional support not low	**0.911**	**0.950**	0.346	**0.841**	**1.000**	0.000
Depression not clinical	**0.874**	**0.930**	0.341	**0.973**	**0.676**	**0.436**
Depression clinical	0.086	0.029	0.278	0.017	0.175	0.206
Depression severe	0.040	0.041	**0.381**	0.010	0.149	0.358
Never homeless	**0.814**	0.299	0.306	**0.667**	0.283	0.252
Past homeless	0.133	**0.556**	**0.396**	0.266	**0.423**	**0.375**
Current homeless	0.053	0.145	0.298	0.067	0.294	0.373
Never incarcerated	**0.970**	0.429	**0.456**	0.391	0.151	0.193
Incarcerated >1 year ago	0.000	**0.468**	0.361	**0.437**	**0.442**	**0.445**
Incarcerated past year	0.030	0.103	0.183	0.172	0.407	0.362

*Highest response probability within each item marked in bold to facilitate interpretation. Numbers of individuals in each class are based on the maximum posterior probability of membership in the baseline (no-covariates) model*.

For men, we again selected the three-class model as optimal based on the Vuong–Lo–Mendell–Rubin likelihood ratio tests of nested models; this solution is presented in Table 3. BIC was lowest for the four-class model among men, but the three-class solution was preferred as it had the highest entropy and was more easily interpreted than the four-class solution. Class 1 was labeled *Personal Assets* (38.0% of men); these men were more likely to have basic needs met (0.758) and to be employed (0.428), less likely to have low education (0.256) and low health literacy (0.070). They also were more likely to have instrumental (0.769) and emotional support (0.841), and were unlikely to have clinically significant (0.017) or severe (0.010) depression. Finally, compared with men in other syndemic classes, men in this class were less likely to be currently homeless (0.067) or recently incarcerated (0.172). Class 2 was labeled *Low Resources/Social Assets* (30.3%); relative to men in the personal assets class, men in this class were more likely to have unmet basic needs (0.617), low education (0.447), to be unemployed (0.862), to have clinically significant (0.175) or severe (0.149) depression, current homelessness (0.294), and recent incarceration (0.407). However, men in this class were likely to have emotional (1.000) and instrumental (0.627) support. Class 3 was labeled *Low Resources and Support* (31.7%); these men had challenges similar to the men in the *Low Resources/Social Assets* class, but also were very unlikely to have emotional (0.000) or instrumental (0.161) support.

Among women, older age increased the odds of membership in the *Low Resources and Support* class relative to the *Assets and Few Historical Risks* class (OR = 1.35; 95% CI: 1.25–1.43). Older age also increased the odds of membership in the *Historical Risk/Assets* class relative to the *Assets and Few Historical Risks* class (OR = 1.34; 95% CI: 1.26–1.44).

Among men, relative to the *Personal Assets* class, older age increased the odds of membership in the *Low Resources/Social Assets* class (OR = 1.19; 95% CI: 1.16–1.23). Older age also increased the odds of membership in the *Low Resources and Support* class relative to the Personal Assets class (OR = 1.18; 95% CI: 1.15–1.22).

### Associations between Syndemic Class Membership and Substance Use Problems

For each sex, past year substance use problem was modeled as a function of age and syndemic profile (Table 4). Among women, older age (OR = 1.02; 95% CI: 1.00–1.03) and membership in either the *Low Resources and Support* (OR = 8.50; 95% CI: 3.85–18.74) or *Historical Risk/Assets* (OR = 2.81; 95% CI: 1.29–6.12) syndemic classes increased the odds of a past year substance use problem relative to the *Assets and Few Historical Risks* class.

**Table 4:** Associations between syndemic class and substance use problem: multivariate logistic regression.

	Adjusted odds ratio	95% CI	*p*-Value
**Female model**			
Age	1.02	1.00–1.03	0.007
Latent class			
Historical risk/assets vs. assets and few historical risks	2.81	1.29–6.12	0.009
Low resources and support vs. assets and few historical risks	8.50	3.85–18.74	<0.001
**Male model**			
Age	1.00	0.99–1.02	0.429
Latent class			
Low resources/social assets vs. personal assets	4.46	2.57–7.75	<0.001
Low resources and support vs. personal assets	11.68	6.91–19.74	<0.001

Among men, relative to the *Personal Assets* class, membership in both the *Low Resources/Social Assets* (OR = 4.46; 95% CI: 2.57–7.75) and *Low Resources and Support* (OR = 11.68; 95% CI: 6.91–19.74) classes increased the odds of a past year substance use problem.

**Figure 1:**
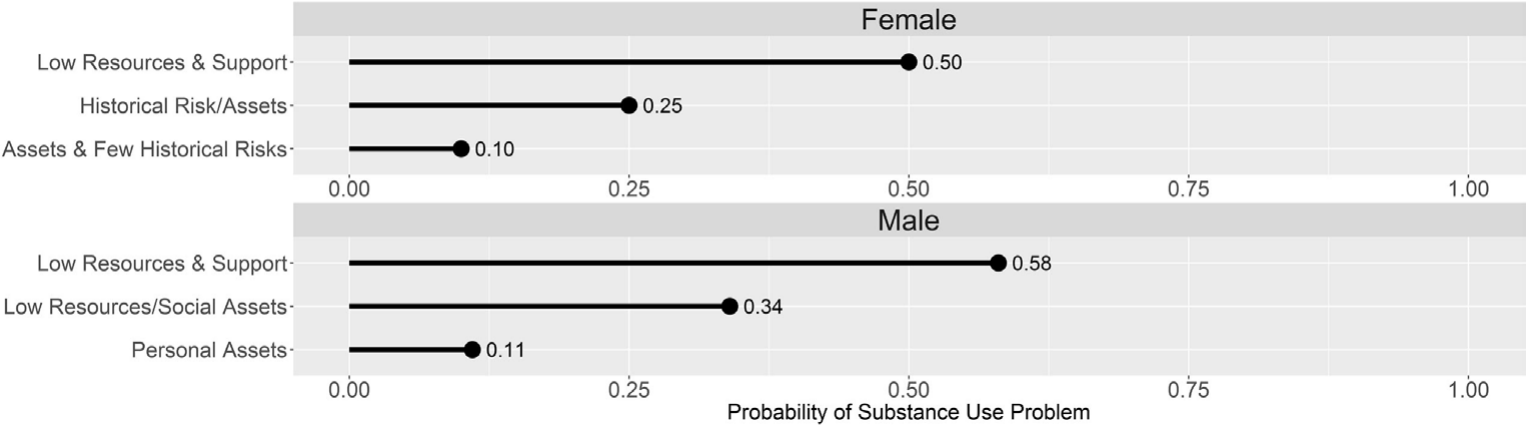
Probability of substance use problem by gender and latent class.

Figure 1 shows the probability of a substance use problem by gender and latent class membership, holding age constant at the mean for each gender. Consistent with Table 4, probabilities of a substance use problem were higher among those in latent classes characterized by more syndemic risk and fewer protective factors. Among women in the *Low Resources and Support* class, 50% evidenced a substance use problem, compared to 10% in the *Assets and Few Historical Risks* class. Among men, 58% of those in the *Low Resources and Support* class had substance use problems, compared to 11% in the *Personal Assets* class.

## Discussion

### Summary

The present study advances the literature on risk and protective factors related to substance use problems among a large population experiencing numerous threats to health and well-being: African-American/Black and Hispanic H-HRA. Within a high-poverty urban context, however, the LCA approach identified patterns of syndemic risk and protective factors among women and men that were associated with the probability of their experiencing a substance use problem in the past year. While all participants were recruited from an urban area with high rates of poverty with elevated rates of numerous serious risk factors at the population level, the latent classes highlight important individual differences within that context, including indications of resilience. For example, among both women and men, a lower risk class was associated with a risk of recent substance use problems that was similar to the rates in the general adult population, which are approximately 8% (60). Moreover, the LCA approach proved useful for simplifying information on risk and protective factors. Before LCA, participants could be in any of 1,728 categories (2^6^ × 3^3^) when all nine syndemic factors were considered together, but the present study yielded much simpler three-class solutions for both women and men that capture the most common combinations of risk factors.

### Gender Differences

Considering syndemic profiles and substance use problem prevalence separately for women and men yielded important insights. Within the high-poverty urban context, men had more risk and fewer protective factors than women, as well as a slightly higher prevalence of substance use problems (38.4 versus 32.1%). The syndemic approach and LCA helped highlight factors underlying those gender differences and the specific confluence of risk factors co-occurring most strongly with substance use problems. In other words, the latent classes helped to explain gender differences in substance use problems by identifying differences in patterns of risk and resilience between women and men.

There were noteworthy differences on syndemic indicators between women and men in similar latent classes. Men in the Personal Assets class were more likely to have a history of homelessness and incarceration and less likely to be employed than women in the Assets and Few Historical Risks class. Women in the Historical Risk/Assets class had more resilience and fewer risk factors than men in the Low Resources/Social Assets class. Specifically, in these moderate risk classes, women were more likely to have basic needs met and instrumental support, and were less likely to have low educational attainment, low health literacy, employment, depression, current homelessness, and recent incarceration than men. In the highest risk class for each gender, women were more likely to have low educational attainment, and less likely to have low emotional support, depression, and recent incarceration than men.

### Risk and Resilience Latent Class Indicators

Both risk and resilience factors were strongly related to latent class membership for both women and men. Resilience factors of instrumental and emotional support and educational attainment helped to differentiate moderate highest risk participants. Among women, the resilience factor of current employment helped to differentiate moderate (historical risk/assets) and lowest (assets and few historical risks) risk participants. Among men, the resilience factors of educational attainment and current employment helped to differentiate the personal assets class from the other two latent classes. These patterns indicate resilience factors can be helpful in measuring syndemic latent classes.

### Latent Classes and Associations with Substance Use Problems among Women

Women evidenced three classes in total, with one class (Assets and Few Historical Risks; 27.1%) comprising the lowest prevalence of risk, along with two higher risk classes. Thus, LCA was useful in uncovering a sub-group of H-HRA women with a relatively modest confluence of risk factors, at least in comparison to their peers in this same population. Importantly, women in the Assets and Few Historical Risks class differed from their peers in that they had never been either homeless or incarcerated, their education level was not poor, and they were employed. In contrast, among the two higher risk classes, Historical Risk/Assets had serious risk factors, namely, homelessness (0.700), incarceration (0.571), and unemployment (0.635), but also protective factors, similar to their peers in the Assets and Few Historical Risks class. However, almost 40% of women fell into the Low Resources and Support class a group experiencing poor outcomes on virtually every index we examined, and almost no evidence of protective factors.

Rates of substance use problems were greatly elevated in the two higher risk classes in comparison to the general population and the lowest risk class of women in the present study. In fact, those in the Low Resources and Support syndemic profile with the fewest protective and most risk factors were more than eight times more likely to exhibit substance use problems than their lower risk peers.

These findings among women, therefore, highlight patterns of both risk and resilience that would not be apparent in studies using other types of analyses, including descriptive statistics. Furthermore, results highlighted the substantial proportion with numerous indicators of resilience and relatively little risk. Although the present study did not examine the timing of events, these findings suggest the importance of historical, structural risk factors such as homelessness and incarceration. Women in the lower risk class had neither of these indicators, while those in the two higher risk groups had substantial rates of both. The protective nature of educational achievement, which was lowest in the Low Resources and Support class, also was evident. Findings also suggest the primacy of social assets in serving as protective factors with respect to substance use problems. Alternately, substance use problems can interfere with social relationships (61), potentially reducing available social support. On the other hand, more than 80% of women had satisfactory health literacy and this domain appears not to be a critical component of syndemics for H-HRA women, perhaps because women’s regular involvement in health services fosters this important skill (62).

### Latent Classes and Associations with Substance Use Problems among Men

Overall, men had somewhat higher rates of substance use problems than women, consistent with the existing literature (63). Profiles of risk and protective factors were similar among men and women, and three separate classes also best described men. A higher proportion of men were in the lowest risk class compared to among women (Personal Assets, 38.0%). Men in this lowest risk class tended to have good educational achievement, less homelessness, a lower risk for incarceration than in the other two classes, more social and instrumental support, and employment was not uncommon—the only class to evidence a relatively high probability of employment. Rates of substance use problems were modest in this lowest risk class and very similar to rates in the general population. Men in the Low Resources and Support class were almost 12 times more likely than their lower risk peers to have a substance use problem. Indeed, this is the only class with low levels of both instrumental and emotional support. Although the cross-sectional nature of the present study does not allow us to determine causal pathways, it does highlight the deleterious nature of low levels of emotional and instrumental support as one important component of syndemics.

### Age and Latent Class

For both men and women, the chances of being found in a higher risk class increases with age, and by the age 40 years, the chances of being in the lowest risk class was small. This suggests the population evidences an age or cohort effect, where younger H-HRA have fewer risk factors, or that risk factors accumulate over time and with age, the latter hypothesis being speculative given the cross-sectional nature of the data.

### Implications

These findings highlight multiple opportunities for the prevention of, and interventions to ameliorate, substance use problems among H-HRA. These include the importance of education, particularly for H-HRA women, and interventions targeting young women at risk for dropout have shown promise (64). Further, they highlight the primacy of homelessness and incarceration as risk factors for poor outcomes, and at the same time, suggest opportunities for substance use prevention and treatment interventions in shelters and criminal justice settings. While substance use problems are treatable, often there are psychosocial and structural barriers to effective treatment (59–61), barriers which may be related to syndemic factors. Women in the Low Resources and Support class and men in the Low Resources and Support classes may benefit from specialized outreach efforts (65, 66) and wrap-around clinical services (67), particularly because they lack emotional and instrumental support, as well as job training programs to address unemployment. Future research on the optimal and most cost-effective combinations of interventions is needed, particularly for this sub-group of women in the Low Resources and Support class and men in the Low Resources and Support class, more than half of whom experience substance use problems.

The syndemic profiles captured in latent classes have implications for the tailoring of substance use treatment and prevention. Among those with a current problem, addressing syndemic factors as part of treatment, or adapting treatments to work more effectively in the presence of syndemic factors, could improve outcomes of substance use treatment. Integrated care that considers multiple problems simultaneously within a single system may lead to better outcomes than care fragmented across multiple, separate systems. Those in classes at higher risk for substance use problems, but without a current problem, may benefit the most from prevention interventions. This suggests syndemic risk and protective factors need to be assessed in a variety of contexts where people may present—jail, homeless shelters, primary care, and others. Addressing modifiable syndemic factors might improve prevention interventions for substance use problems.

### Limitations

Because all interview questions were asked at the same time, we do not know how syndemic factors and substance use problems developed over time. It is not clear whether chronic struggles with substance use came before and led to the absence of protective and presence of risk factors defining the syndemic profiles. While the cross-sectional study design does not allow causal inferences, syndemic factors, and substance use problems are strongly associated. Rather than thinking of syndemic profiles as preceding and causing substance use problems, substance use problems may be an additional indicator of syndemic profile membership, as the identified latent classes, both for women and men, had substantially different probabilities of substance use problems. Longitudinal research which considers transitions in syndemic profiles and substance use problems is needed to make causal inferences. Indicators of syndemic classes did not include childhood trauma, intimate partner or other violent victimization, family strengths, or engagement with community institutions.

These findings are limited by the specific measures of syndemic factors and substance use problems employed. The psychosocial construct of emotional support was measured with a single interview item. While coherent associations between latent classes and this crude measure of emotional support were found, a more comprehensive assessment of emotional support might yield additional insights into how this construct relates to syndemic classes and health outcomes such as substance use problems. Also, the specific measures of risk and resilience included are not comprehensive. Additional measures of risk and resilience, including constructs measured at individual, social, and structural levels, might yield additional insights.

## Conclusion

Latent class analysis revealed a small number of syndemic profiles for women and men in a high-poverty urban area. These syndemic profiles were strongly related to prevalence of a current substance use problem. Profiles with more risk and fewer resilience factors were associated with substance use problems, but profiles with fewer risk and more resilience factors had rates of substance use problems that were very similar to the general adult population. Addressing syndemic factors in substance use treatment and prevention may yield improved outcomes.

## Ethics Statement

The study was approved by the New York University School of Medicine Institutional Review Board. All participants gave written informed consent in accordance with the Declaration of Helsinki.

## Author Contributions

MG conceived of the overall study concept and design. CC participated in the design of the study. CC and SL planned statistical analyses, and CC conducted statistical analyses. CC, MG, SL, and SV drafted the manuscript. All authors read and approved the final manuscript.

## Conflict of Interest Statement

The authors declare that the research was conducted in the absence of any commercial or financial relationships that could be construed as a potential conflict of interest. The reviewer, JH, and the handling editor declared their shared affiliation, and the handling editor states that the process nevertheless met the standards of a fair and objective review.
